# Clinical Case of Comorbid Course of Metabolically Associated Fatty Liver and Pancreas Disease in a Child with Prader-Willi Syndrome

**DOI:** 10.34763/jmotherandchild.20252901.d-25-00015

**Published:** 2025-11-05

**Authors:** Tetyana O. Kryuchko, Inna M. Nesina, Svitlana I. Lytus, Olha A. Poda, Liudmyla M. Bubyr

**Affiliations:** Department of Paediatrics No. 2, Poltava State Medical University. Address: St. 23 Shevchenko Street, Poltava, 36011, Ukraine.

**Keywords:** metabolically associated fatty liver disease, metabolically associated fatty pancreas disease, metabolically associated steatotic hepatitis, steatosis, obesity, Prader-Willi syndrome, exocrine pancreatic insufficiency

## Abstract

Prader-Willi syndrome is the most common form of genetic obesity in children. The aim of our study was to analyse the progressive course of metabolically associated steatotic liver disease along with the development of pancreatic exocrine function deficiency in Prader-Willi syndrome.

The presented clinical case is an example of the early development of metabolic steatotic liver disease in a child with Prader-Willi syndrome, complicated by liver fibrosis and exocrine pancreatic insufficiency. This clinical case is interesting because the patient developed signs of exocrine pancreatic insufficiency at an early age in the form of a decrease in pancreatic elastase in the stool — a consequence of fatty tissue replacement, i.e., the development of pancreatic steatosis. Therefore, the efforts of the treatment protocol are focused on a multidisciplinary approach including the examination of liver and pancreatic function. This allows for control of the progression of the disease, and reduces the risk of obesity-related complications. Despite the rarity of such cases, physicians should be alert in managing these patients.

## Introduction

Childhood obesity remains a global problem despite a large number of studies. One of the most common genetic forms of obesity is Prader-Willi syndrome (PWS), which is a complex neurogenetic, endocrine and neurobehavioural disorder that occurs when an inherited imprinted gene on chromosome 15q11.2–q13.1 is not expressed. It may be the result of a paternal deletion, maternal uniparental disomy of chromosome 15, or an imprinting defect. One of the complications in children with PWS is obesity. This complication requires special attention, as it significantly affects quality of life and can lead to a number of comorbidities, including the development of metabolic diseases. Obesity is the main modifiable risk factor for insulin resistance in children and adolescents [4].

## Case report

A boy, M. (8 years old), born at 36 weeks of gestation, with a body weight of 2,120 g, height 47 cm, by caesarean section, was under our observation. The pregnancy was complicated: ARVI (week 13), foetal growth retardation syndrome (week 32), and prematurity. During the examination in the maternity hospital, the following were found: neonatal hypoglycaemia (blood glucose level: 1.8-3.0-2.5 mmol/l), left-sided cryptorchidism, torticollis, partial syndactyly of two to three toes of the left foot, and mandibular hypoplasia. Since birth, muscle hypotonia has been observed, but the boy began to hold his head up at two months, sit at seven months, and stand at eight months. During the first year of life, the child gained 6 kg and 380 g.

The medical history shows that the above complaints had been noted since the boy began to gain weight rapidly at the age of two and his parents consulted an endocrinologist. During the initial hospitalisation in a specialised department, biochemical blood tests revealed elevated levels of cytolytic syndrome and carbohydrate profile (Table 1). After genetic and molecular confirmation, the boy was diagnosed with Prader-Willi syndrome (46, XY ish del (15)q11-q13 D15-1, D15s10-Pm (+)), with neuroendocrine obesity, progressive course, insulin resistance, metabolically associated steatotic liver disease, delayed psycholinguistic development, and kyphoscoliotic posture.

**Table 1.** Laboratory examination.

**Item**	**Results**	**Reference range**
2 years		

ALT	141 U/L	˂41 U/L
AST	112 U/L	˂ 37 U/L
Insulin level	19.0 mIU/mL	2.3–26.4 mIU/mL
HOMA index	4.3 U	˂2.5 U

4 years		

ALT	233 U/l	˂41 U/L
AST	186 U/l	˂ 37 U/L

5 years		

ALT	543 U/l	˂41 U/L
AST	344 U/l	˂ 37 U/L
Insulin level	40.9 mIU/mL	2.3–26.4 mIU/mL
HOMA index	9.3 U	˂2.5 U

7 years		

ALT	7.13 ukat/l	˂ 0.83 ukat/l
AST	3.57	˂ 0.85
Pancreatic fecal elastase	43.0 mcg/g	˃ 200 mcg/g

8 years		

ALT	352 U/l	˂41 U/L
AST	134 U/l	˂ 37 U/L
Insulin level	25.7 mU/l	0.3–26.4 mIU/ml
HOMA index	3.9 U	˂ 2.5 U
Pancreatic faecal elastase	108.56 mcg/g	˃200 mcg/g

At the age of four, the boy first visited a paediatric gastroenterologist with complaints of periodic liquid stools with impurities of undigested food, along with increased appetite, being overweight, and delayed psychomotor development.

Examination in the gastroenterology department showed increasing indicators of mesenchymal inflammatory syndrome (Table 1). and pathological changes in the coprogramme — the presence of creatorrhoea with steatorrhoea. Transient liver elastometry confirmed the presence of fibrotic changes (the average indicator: 6.42 kPa out of 10 measurements), with a 0.95 degree of fibrosis according to the METAVIR-F1 system.

A year later, an increase in transaminases and basal insulin levels indicated a high degree of inflammation in the liver and parenchyma (Table 1). The progression of insulin resistance was confirmed, along with further development of metabolic-associated steatotic liver disease with transition to steatohepatitis. For the purpose of regression of insulin resistance and enzymatic markers of cytolysis — which have a direct impact on the development of steatohepatitis — the boy was prescribed combined courses of hepatoprotective drugs [2].

As a result of the Russian-Ukrainian war, the child was forced to leave for Germany for security reasons [6]. When medical care was sought for him there, further progression of metabolic-associated steatotic liver disease, along with the development of severe exocrine pancreatic insufficiency, was established (Table 1).

When admitted to hospital in 2024, the child complained of weight gain (3 kg and 500g over the prior two months), recurrent diarrhoea after a dietary disorder, leg muscle pain, and delayed psycholinguistic development. An objective examination revealed the formation of characteristic phenotypic signs of Prader-Willi syndrome: almond-shaped optic gaps, narrow nasal bridge, thin upper lip with downwardly curved corners of the mouth, partial syndactyly of two to three toes of the left foot and a BMI of 34 kg/m^2^. Despite periodic courses of symptomatic therapy for four years, usually with the ultimate goal of normalising serum aminotransferases or reducing/eliminating steatosis according to sonography, the boy had progressive fibrotic liver changes. This was confirmed by transient elastometry with an average indicator of 7.38 kPa, SDev 1.07, which corresponds to the fibrosis stage according to the METAVIR F2 system. The results of the patient’s examination demonstrate that the accumulation of fatty acids in hepatocytes leads to defects in the insulin signalling pathway in individuals with genetic susceptibility, and this is reflected in the imbalance of carbohydrate metabolism (Table 1). Complex therapy slightly reduced the activity of hepatocyte cytolysis, but its markers remained elevated (Table 1).

The patient’s results confirm that even a short course of genetic obesity also affects the indicators of exocrine pancreatic insufficiency: a decrease in pancreatic elastase (108.56 mcg/g, compared to the normal ˂ 200 mcg/g) and changes in the coprogramme (the presence of muscle fibres, neutral fat and fatty acids). According to current knowledge, low serum levels of vitamin D (D25 hydroxyvitamin D: 60.5 nmol/l, vs. reference values of 75–125 nmol/l) are associated with the development of metabolic steatohepatitis.

In order to influence the clinical course of comorbid pathology, correct the functional parameters of the mesenchymal inflammatory syndrome, and prevent the progression of steatotic disease, the boy was recommended to adhere strictly to a diet, limit his consumption of easily digestible carbohydrates and fats, and engage in physical activity. He was also prescribed a course of drug therapy (vitamin D, ursodeoxycholic acid, arginine citrate/betaine and enzyme preparations) to help restore cell membranes and stimulate the processes of fat oxidation and utilisation, thus reducing fatty liver, slowing down collagen formation in the liver, and reducing inflammatory reactions. Enzyme preparations are indicated to improve the exocrine function of the pancreas. The boy continues to follow a dietary regimen and engage in light physical activity for up to 60 minutes a day, which distracts him from frequent meals and helps him lose weight.

## Discussion

Despite the fact that the complications described in patients with PWS are not very common, especially in childhood, the clinical case presented clearly demonstrates the individual characteristics of the course of Prader-Willi syndrome. It is well-known that obesity is the cause of metabolic complications and poor quality of life in children with PWS [5].

In the case presented here, it was only after complaints of a sharp increase in the boy’s body weight that a diagnostic search for genetically-determined obesity began. However, the assessment of height and weight is an important component that characterises the harmonious development of a child. Symptoms of muscle hypotension, hypoglycaemia, decreased weight gain, and stigmatic abnormalities are characteristic signs of genetic diseases and a valuable cue to start diagnostic testing.

This clinical case also demonstrated the relationship between the steatotic process in the liver and metabolic changes in the pancreas in a child with a short history of the disease. Modern research by Kweh and colleagues [7] helped to characterise and improve understanding of the phases of nutrition in PWS. During phases 1a and 1b, plasma insulin levels remain normal; the transition from phase 1b to phase 3, however, is accompanied by a significant increase in insulin, coinciding with hyperphagia, weight gain and obesity. Increases in fasting plasma insulin levels and HOMA index indicate the development of hepatic insulin dysfunction [7].

Modern scientific research in this area helps improves understanding of the complex relationship between diet, body, intestinal microbiota, and the development of severe complications. An analysis of the boy’s glycaemic profile showed that from the age of two to eight years, he had persistent hyperinsulinaemia, which may be confirmatory evidence that the patient was in the 2nd phase of PWS nutrition. At the moment, insulin levels and the HOMA index have slightly decreased, but signs of exocrine pancreatic insufficiency have appeared in the form of a decrease in pancreatic elastase in the faeces — a consequence of further fatty tissue replacement, i.e., the development of pancreatic steatosis. The development of pancreatic exocrine function deficiency in PWS is quite rare and significantly worsens the patient’s prognosis. Therefore, the efforts of the treatment protocol are focused on stabilising the child’s condition and transitioning to the fourth phase of nutrition, which will have a positive effect on metabolic inflammation of the liver and the functional capacity of the pancreas.

The clinical course of PWS clearly shows that lifestyle interventions are crucial in the treatment of these patients. A controlled and balanced nutritional programme should be implemented from the first months of life, and the patient should be closely monitored on an ongoing basis [8]. The diet for children with PWS should include a daily energy intake restriction of up to 60%; the usual diet for healthy people of the same age is thus not suitable for PWS patients [9]. Treatment of PWS should also include adequate physical activity, such as swimming, brisk walking, running, aerobic and strength exercises, or cycling for at least 60–90 minutes per day.

A multidisciplinary approach and routine check-ups help to control the course of the disease and reduce the risk of obesity-related complications. Lifestyle modification, along with the development of personalised regimen of diet, medication and physical activity, will help prevent the progression of comorbidities in children with both genetic and phenotypic variants of obesity.
